# Associations of Cannabis Use, High-Risk Alcohol Use, and Depressive Symptomology with Motivation and Attempts to Quit Cigarette Smoking Among Adults: Findings from the 2020 ITC Four Country Smoking and Vaping Survey

**DOI:** 10.1007/s11469-023-01214-8

**Published:** 2023-12-14

**Authors:** Shannon Gravely, Pete Driezen, Lion Shahab, Erin A. McClure, Andrew Hyland, K. Michael Cummings, Katherine A. East, Gary C. K. Chan, Hannah Walsh, Neal L. Benowitz, Coral E. Gartner, Geoffrey T. Fong, Anne C. K. Quah, Danielle M. Smith

**Affiliations:** 1 International Tobacco Control Policy Evaluation (ITC) Project, Department of Psychology, University of Waterloo, Waterloo, Canada; 2 School of Public Health Sciences, University of Waterloo, Waterloo, Canada; 3 Department of Behavioural Science & Health, University College London, London, UK; 4 SPECTRUM Research Consortium, The University of Edinburgh, Edinburgh, UK; 5 Hollings Cancer Center, Medical University of South Carolina, Charleston, SC USA; 6 Department of Psychiatry and Behavioral Sciences, Medical University of South Carolina, Charleston, SC USA; 7 Roswell Park Comprehensive Cancer Center, Buffalo, NY USA; 8 National Addiction Centre, Institute of Psychiatry, Psychology and Neuroscience, King’s College London, London, UK; 9 National Centre for Youth Substance Use Research, The University of Queensland, Brisbane, Australia; 10 University of California San Francisco, San Francisco, CA USA; 11 School of Public Health, Faculty of Medicine, The University of Queensland, Brisbane, Australia; 12 Ontario Institute for Cancer Research, Toronto, Canada

**Keywords:** Cigarette smoking, Cannabis, Alcohol, Depressive symptomology, International, Public health

## Abstract

This study assessed independent and interaction effects of the frequency of cannabis use, high-risk alcohol use, and depressive symptomology on motivation and attempts to quit cigarette smoking among adults who regularly smoked. Cross-sectional data are from the 2020 International Tobacco Control Four Country Smoking and Vaping Survey and included 7044 adults (ages 18 + years) who smoked cigarettes daily in Australia (*n* = 1113), Canada (*n* = 2069), England (*n* = 2444), and the United States (USA) (*n* = 1418). Among all respondents, 33.1% of adults reported wanting to quit smoking “a lot,” and 29.1% made a past-year quit attempt. Cannabis use was not significantly associated with either outcome (both *p* ≥ 0.05). High-risk alcohol use was significantly associated with decreased odds of motivation to quit (*p* = 0.02) and making a quit attempt (*p* = 0.004). Depressive symptomology was associated with increased odds for both outcomes (both *p* < 0.001). There were no significant 2- or 3-way interactions between cannabis use, alcohol consumption, and depressive symptomatology. Overall, just over a quarter of adults who smoked daily reported making a recent quit attempt, and most were not highly motivated to quit. Longitudinal research should investigate whether there are linkages between cannabis use, risky alcohol consumption, and/or depression on successful long-term smoking cessation.

**Supplementary Information:**

The online version contains supplementary material available at 10.1007/s11469-023-01214-8.

More than 7 million people die each year from tobacco use (WHO, 2022)—far more than from alcohol and illicit drug use combined (GBD, 2020). Nicotine dependence is a chronic and relapsing condition, and many people who smoke tobacco are dependent on nicotine (USHHS, 2020). Greater nicotine dependence has been found to be associated with lower motivation to quit smoking, greater difficulty in quitting, and quitting failure (USHHS, 2020; WHO, 2010).

There is well-established literature demonstrating that certain subpopulations more commonly smoke cigarettes compared to the general population, including those who regularly use cannabis (AHS, 2021; Chu et al., 2023; Fix et al., 2019; Goodwin et al., 2018; Leos-Toro et al., 2018; Statistics Canada, 2018; Strong et al., 2018), people who engage in risky alcohol use (McKee et al., 2007; Weinberger et al., 2017, 2019), and those with poorer mental health, including depression (Fergusson et al., 2003; Fluharty et al., 2017; Johnson et al., 2006; Kock et al., 2023; Lipari and Horn, 2017; Prochaska et al., 2017; Sharma et al., 2016; WHO, 2021). While there is evidence that these factors are independently associated with higher cigarette smoking prevalence, there are three important gaps in the literature: (1) mixed evidence regarding if these factors are associated with motivation to quit smoking and attempt to quit smoking, particularly for cannabis use, (2) evaluating the interaction between these three factors on quit motivation and quit attempts, and (3) cross-country comparisons based on varying regulatory environments, cultural norms, treatment/health care systems, inter alia.

Several epidemiological studies indicate that cannabis use is associated with greater nicotine dependence and a lower likelihood of trying to quit smoking and successfully quitting smoking relative to those who do not use cannabis (Driezen et al., 2022; Goodwin et al., 2022; Strong et al., 2018; Voci et al., 2020; Weinberger et al., 2018, 2020). In contrast, secondary analyses of randomized smoking cessation trials have generally found no significant differences in smoking cessation rates between people who use and do not use cannabis (Hendricks et al., 2012; McClure et al., 2018, 2020; Metrik et al., 2011; Rabin et al., 2016; Walsh et al., 2021). A recent cross-sectional study by Gravely et al. examined differences between adults who smoked cigarettes daily and who did and did not co-use cannabis in Australia, Canada, England, and the USA, and found cannabis use (vs. no use) to be unrelated to several cigarette smoking-related measures, including urges to smoke, dependence, plans to quit smoking, and perceptions about their level of addiction to cigarettes (Gravely et al., 2022).

There are well-established associations between heavy alcohol consumption and nicotine dependence, and a lower likelihood of successful smoking cessation (Hughes & Kalman, 2006; Kahler et al., 2010; Mendelsohn, 2016; Toll et al., 2012, 2015; Amsterdam and Brink, 2023; Yimsaard et al., 2023), although people who are dependent on alcohol appear to be as motivated to quit smoking as those who smoke in the general population (Mendelsohn, 2012). Similarly, while the relationship between mental illness, especially depression, and difficulty quitting smoking is complex, evidence suggests that adults who smoke cigarettes and have depression are highly motivated to quit smoking (Cooper et al., 2016; Haukkala et al., 2000; Kastaun et al., 2022; Lembke et al., 2007; Mendelsohn, 2012; Siru et al., 2009; Yimsaard et al., 2023), but are less successful in maintaining abstinence and/or recovering after a relapse compared to those without mental health conditions (Cooper et al., 2016; Hitsman et al., 2013; Johnson et al., 2006; Muench et al., 2020; Ranjit et al., 2020).

Cannabis use, risky alcohol use, and depression have all been studied separately to understand their effects on tobacco use outcomes, but the possible interactions among these co-occurring behaviors and conditions on smoking is under-researched. Notably, those who regularly use cannabis and who engage in heavy alcohol use and/or binge drink are more likely to have depression (and vice versa) (Bellos et al., 2013; Boden & Fergusson, 2011; Feingold & Weinstein, 2021; Gunn et al., 2018; Hindocha et al., 2021; Konefal et al., 2019; Meyer and Leece, 2018; Pacek et al., 2020; Paljärvi et al., 2009; Peters et al., 2012, 2014; Yurasek et al., 2017), but little is known about whether these factors are collectively associated with lower motivation to quit smoking and/or reduced attempts to quit.

In addition to assessing populations who live in one jurisdiction or country, our study presents a unique opportunity to examine these relationships across and within several countries. At this time, little is known about differences or similarities between countries as to whether adults who regularly smoke cigarettes are less motivated or make fewer attempts to quit smoking among those with co-occurring substance use and depressive symptomology. As countries have differing cannabis and alcohol regulatory environments, rates of cannabis use, and cannabis co-use practices (e.g., mixing cannabis and tobacco vs. using them separately) (Gravely et al., 2020), presenting data both across and within countries is warranted. To our knowledge, no studies have tested these linkages across multiple countries. Therefore, we conducted an international study among adults who smoked tobacco cigarettes daily in 2020, and resided in Australia, Canada, England, or the USA, and assessed (1) the association of frequency of cannabis use, high-risk alcohol use, or depressive symptomology on motivation to quit cigarette smoking and attempts to quit smoking, overall and stratified by each country, (2) whether high motivation to quit was associated with making a recent quit attempt, and (3) higher-order interaction effects between the frequency of cannabis use, high-risk alcohol use, and depressive symptomology on each of the outcomes. We also tested country interaction effects with the independent variables.

## Methods

### Study Design, Procedure, and Sample

The International Tobacco Control Policy Evaluation Project Smoking and Vaping (ITC 4CV) Survey is a cohort study conducted in Canada, the USA, England, and Australia. Adults (≥ 18 years) who currently smoke (≥ monthly), recently quit smoking (quit ≤ 2 years), and/or currently vape nicotine (≥ weekly) were recruited from online commercial panels. All eligible respondents provided consent and completed an online survey. Study procedures and materials were approved by research ethics boards in all countries. Further study details are reported elsewhere (ITC Project, 2021).

**Fig. 1:**
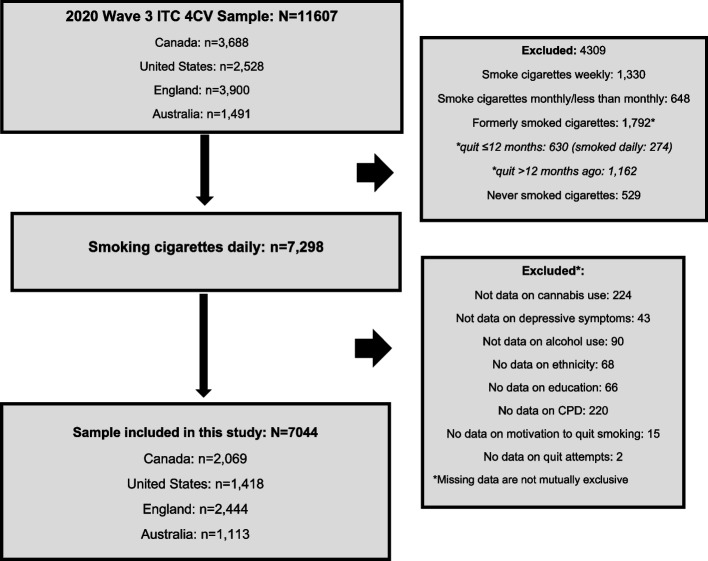
The sample selection process

Cross-sectional data for this study were from the ITC 4CV Wave 3 (2020) Survey (conducted from February to June 2020). Respondents included those who were retained from previous survey waves (Wave 1: 2016 and/or Wave 2: 2018, *n* = 3228, 45.8%) and new respondents who were recruited to replace those lost to attrition (*n* = 3816, 54.2%). Respondents who were initially considered for inclusion were those who were currently smoking cigarettes daily and provided complete data about their cannabis and alcohol use, depressive symptomology, and the outcome measures (motivation to quit smoking and quit attempts). Those who reported already having quit smoking at the time of the Wave 3 survey, and previously smoked daily in the past 24 months, were excluded (*n* = 274). Figure 1 shows the sample selection process for this study.

### Measures

#### Outcome Variables

##### Motivation to Quit Smoking Cigarettes

All respondents were asked “How much do you want to quit smoking?” Response options included “not at all,” “a little,” “somewhat,” “a lot,” or “don’t know.” This outcome was dichotomized as “a lot” (high motivation to quit) vs. “otherwise” (all other responses).

##### Recent Attempt to Quit Smoking Cigarettes

Respondents were asked whether they had made an attempt to quit smoking in the last two years and, among those who reported making an attempt, how long ago they did so. Those who made a quit attempt in the last 12 months were considered to have made a ‘recent’ attempt to quit smoking and was categorized as: “yes” (made an attempt to quit smoking in the past year) vs. “no/don’t know”.

#### Independent Variables

##### Cannabis Use

All respondents were asked: “When was the last time you used marijuana/cannabis?” Response options included “never used cannabis,” “in the last 30 days,” “in the last 1–12 months,” and “more than a year ago.” If respondents self-reported cannabis use in the last 12 months, they were asked a follow-up question: “On average, how often do you CURRENTLY use any form of marijuana/cannabis?” Response options included “daily,” “weekly,” “monthly,” “occasionally (less than monthly),” and “I have quit using it.” Based on above two survey questions, respondents were categorized into one of four groups: (1) “daily cannabis use”; (2) “less than daily, but at least weekly/monthly cannabis use”; (3) “less than monthly (occasional) use and used it in the last year”; or (4) “no current cannabis use” (never used/quit using it/have not used it in the last year).

We did not exclude any type of cannabis product used in the last 12 months, but the majority of respondents reported using the smoked form (88%). Other forms of cannabis use included edibles (26.8%), used it orally (13.0%), vaped it in a liquid (12.9%), dabbed concentrates (11.3%), and/or vaporized dried leaf/bud (9.0%). These responses were not mutually exclusive (respondents could have used multiple forms). Those who declined to answer the questions about cannabis use were excluded (*n* = 224).

##### Alcohol Use

All respondents were first asked “How often do you have a drink containing alcohol?” Follow-up questions assessed frequency of alcohol consumption on a typical day of drinking and how often they binge drink (more than 6 drinks on one occasion). Alcohol consumption was defined using the *National Institute on Alcohol Abuse and Alcoholism’s* definition (NIAAA), where levels of alcohol use are defined as “low risk” (≤ 4 drinks per occasion for men, ≤ 2 drinks per occasion for women) and “high risk” (≥ 5 drinks per occasion for men, ≥ 3 drinks per occasion for women, and ≥ 6 drinks on a single occasion at least once a month for men and women). We compared those who self-reported high-risk alcohol use based on the above criteria to those who did not (low risk or no use). Those who declined to answer or reported that they did not know how often they consumed alcohol were excluded (*n* = 90).

##### Depressive Symptomology

All respondents were screened for past 30-day depressive symptomology using the two-question case finding instrument (TQI) for depression. The TQI is a useful measure for detecting depression in primary care and has similar test characteristics to other case-finding instruments, including the Center for Epidemiologic Studies Depression Scale and Beck Depression Inventory (Whooley et al., 1997).

The TQI uses two questions to assess depressive symptoms: (1) “During the last 30 days, have you often been bothered by little interest or pleasure in doing things?” (2) “During the last 30 days, have you often been bothered by feeling down, depressed, or hopeless?” Respondents who reported “yes” to both questions were classified as screening positive for depressive symptoms. Those who declined to answer were excluded (*n* = 43).

#### Covariates

##### Cigarettes Smoked Per Day (CPD)

Respondents were asked *“*On a typical day, how many cigarettes do you usually smoke each day, including both [factory-made/packet] and roll-your-own cigarettes?” CPD was categorized as “1–10,” “11–20,” or “21 + ” for analyses. Those who declined to answer or reported that they did not know were marked as missing.

##### Sociodemographic Measures

Sociodemographic measures were age (18–39 vs. 40 +), sex at birth (male, female), income (low, moderate, high, not reported), and education (low, moderate, high). The definitions of “low,” “moderate,” and “high” for income and education can be found under Table 1. Data on race/ethnicity were also collected in Canada, England, and the USA as per each country’s national Census (e.g., White, Black, Indian, Chinese); however, Australia does not collect data on race and ethnicity. Instead, the Census in Australia defines people as “English-speaking” (vs. does not speak English in the home) and whether they are of Aboriginal or Torres Strait Islander background. In line with this, we have defined this variable as “White/English-speaking” vs. “other race or ethnicity/non-English speaking.”

**Table 1:** Characteristics of the study sample (unweighted)

	Canada *n* = 2069	USA *n* = 1418	England *n* = 2444	Australia *n* = 1113	All *N* = 7044
	*n (%)*	*n (%)*	*n (%)*	*n (%)*	*n (%)*
Wave of recruitment					
Wave 1 (2016)	571 (27.6)	424 (29.9)	591 (24.2)	383 (34.4)	1969 (28.0)
Wave 2 (2018)	328 (15.9)	328 (23.1)	389 (15.9)	214 (19.2)	1259 (17.9)
Wave 3 (2020)	1170 (56.6)	666 (47.0)	1464 (59.9)	516 (46.4)	3816 (54.2)
Age					
18–39	865 (41.8)	505 (35.6)	1053 (43.1)	184 (16.5)	2607 (37.0)
40 +	1204 (58.2)	913 (64.4)	1391 (56.9)	929 (83.5)	4437 (63.0)
Sex					
Female	1100 (53.2)	752 (53.0)	1172 (48.0)	546 (49.1	3570 (49.3)
Male	969 (46.8)	666 (47.0)	1272 (52.1)	567 (50.9)	3474 (50.7)
Income					
Low	609 (29.4)	530 (37.4)	534 (21.9)	321 (28.8)	1994 (28.3)
Moderate	580 (28.0)	406 (28.6)	839 (34.3)	239 (21.5)	2064 (29.3)
High	759 (36.7)	478 (33.7)	932 (38.1)	487 (43.8)	2656 (37.7)
Not reported	121 (5.9)	4 (0.3)	139 (5.7)	66 (5.9)	330 (4.7)
Education					
Low	642 (31.0)	557 (39.3)	347 (14.2)	342 (30.7)	1888 (26.8)
Moderate	920 (44.5)	574 (40.5)	1331 (54.5)	462 (41.5)	3287 (46.7)
High	507 (24.5)	287 (20.2)	766 (31.3)	309 (27.8)	1869 (26.5)
Ethnicity/race					
White/English-speaking*	1738 (84.0)	1036 (73.1)	2211 (90.5)	1003 (90.1)	5988 (85.0)
Black/other minority group	331 (16.0)	382 (26.9)	233 (9.5)	110 (9.9)	1056 (15.0)
CPD					
1–10	965 (47.8)	666 (48.4)	1146 (48.6)	367 (33.4)	3144 (45.9)
11–20	797 (39.5)	552 (40.2)	1021 (43.3)	491 (44.6)	2861 (41.7)
21 +	258 (12.8)	157 (11.4)	193 (8.2)	242 (22.0)	850 (12.4)
Years smoking daily					
< 1 year	164 (8.0)	105 (7.5)	135 (5.5)	47 (4.2)	451 (6.4)
1–10 years	577 (28.0)	376 (26.7)	730 (29.9)	157 (14.1)	1840 (26.2)
> 10 years	1323 (64.1)	927 (65.8)	1573 (64.5)	907 (81.6)	4730 (67.4)
Frequency of cannabis use					
Daily	411 (19.9)	207 (14.6)	174 (7.1)	56 (5.0)	848 (12.0)
Weekly/monthly use	309 (14.9)	147 (10.4)	183 (7.5)	65 (5.8)	704 (10.0)
< monthly	207 (10.0)	94 (6.6)	113 (4.6)	56 (5.0)	470 (6.7)
None	1142 (55.2)	970 (68.4)	1974 (80.8)	936 (84.1)	5022 (71.3)
Depressive symptomology					
Yes	663 (32.0)	320 (22.6)	670 (27.4)	261 (23.5)	1914 (27.2)
No/don’t know	1406 (68.0)	1098 (77.4)	1774 (72.6)	852 (76.6)	5130 (72.8)
Level of risk for alcohol consumption					
High-risk	499 (24.1)	265 (18.7)	690 (28.2)	293 (26.3)	1747 (24.8)
Low-risk	902 (43.6)	470 (33.2)	1102 (45.1)	433 (38.9)	2907 (41.3)
None	668 (32.3)	683 (48.2)	652 (26.7)	387 (34.8)	2390 (33.9)

Data are unweighted and unadjusted. Annual household income is defined as “low” (CA: < CAD $30,000; USA: < USD $30,000; EN: < £15,000), “moderate” (CA: CAD $30,000–59,000; USA: USD$30,000–59,000; EN: £15,000–30,000), “high” (CA: ≥ CAD $60,000; USA: ≥ USD $60,000; AU: ≥ AUD $60,000; EN: > £30,000), and “not reported”; education is defined as “low” (all countries: ≤ high school), “moderate” (CA: trade school, community college, some university (no degree); USA: trade school, community college, associate degree, or some university (no degree) EN: further education/ training college below degree level or some university (no degree), “high” (all countries: university degree or post-graduate degree), and “not reported.” Current frequency of cannabis use: (1) “daily cannabis use”; (2) “less than daily, but at least weekly/monthly cannabis use”; (3) “less than monthly (occasional) use and used it in the last year”; or (4) “no current cannabis use” (never used/quit using it/have not used it in the last year). Alcohol consumption was defined using the National Institute on Alcohol Abuse and Alcoholism’s definition (NIAAA), where levels of alcohol use are defined as “low risk” (≤ 4 drinks per occasion for men, ≤ 2 drinks per occasion for women) and “high risk” (≥ 5 drinks per occasion for men, ≥ 3 drinks per occasion for women, and ≥ 6 drinks on a single occasion at least once a month for men and women). All respondents were screened for past 30-day depressive symptomology using the two-question case finding instrument (TQI) for depression. Respondents who reported “yes” to both questions were classified as screening positive for depressive symptoms. *English is the primary language spoken in the home in Australia

### Statistical Analysis

Unweighted descriptive statistics were used to describe the study sample, overall and by country (Table 1). All other analyses were conducted on weighted data. Sampling weights were computed to adjust for the oversampling of some sub-populations, non-response, and other sources of bias. The weights for 4CV3 were designed to make the sample as representative as possible of the adult smoking population in each country with respect to sex, age group, educational attainment, and geography. A *raking algorithm* was used to calibrate cross-sectional sampling weights on smoking status, geographic region, and demographic measures (Kolenikov, 2014). National surveys were used as benchmarks to calibrate the survey weights (ITC Project, 2021). All analyses were conducted using SAS Version 9.4. Statistical significance and confidence intervals were computed at the 95% confidence level using two-tailed tests.

**Fig. 2:**
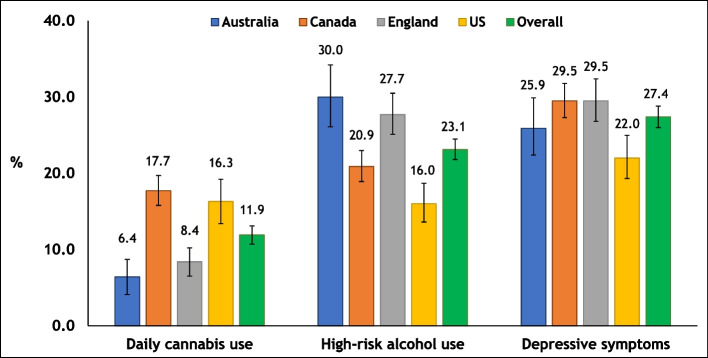
Prevalence of daily cannabis use, depressive symptomology, and high-risk alcohol use among adults who smoke cigarettes daily in Australia, Canada, England, and the USA in 2020. Conditional estimates are descriptive and weighted

In the first set of analyses, weighted descriptive statistics (“proc surveyfreq”) were used to derive conditional estimates for daily cannabis use, high-risk alcohol use, and depressive symptomology, overall and by country (Fig. 2). Second, unadjusted (weighted) logistic regression models were used to assess the association of each of the independent variables (frequency of cannabis use, high-risk alcohol use, and depressive symptomology) with motivation to quit smoking cigarettes and making an attempt to quit cigarette smoking in the past 12 months (Table 2).

**Table 2:** Unadjusted (weighted) regression analyses examining the association between cannabis use, high-risk alcohol use and depressive symptomology on motivation to quit smoking and attempts to quit smoking

Independent variables	High Motivation to quit smoking			Attempted to quit smoking		
	*% (95% CI)*	*OR (95%CI)*	*p-value*	*% (95% CI)*	*OR (95%CI)*	*p-value*
Current frequency of cannabis use			0.88			0.04
Daily	34.2 (29.9–38.7)	1.03 (0.83–1.27)	0.82	31.6 (27.5–35.9)	1.13 (0.91–1.40)	0.26
Weekly/monthly use	32.1 (27.4–37.2)	0.93 (0.74–1.18)	0.57	36.2 (31.3–41.4)	1.39 (1.10–1.76)	0.01
< monthly use (occasional)	32.1 (27.0–37.7)	0.94 (0.72–1.21)	0.61	31.6 (26.5–37.2)	1.13 (0.87–1.47)	0.36
Not using cannabis	33.6 (31.9–35.4)	Reference		29.0 (27.4–30.7)	Reference	
High-risk alcohol use			0.004			0.26
Yes	29.3 (26.3–32.5)	0.78 (0.66–0.92)	0.004	28.5 (25.6–31.7)	0.91 (0.77–1.07)	0.26
Low/none	34.7 (33.0–36.4)	Reference		30.6 (29.0–32.2)	Reference	
Depressive symptomology			< 0.001			< 0.001
Yes	38.9 (36.0–41.9)	1.40 (1.20–1.62)	< .001	37.4 (34.5–40.4)	1.59 (1.37–1.85)	< 0.001
No/don’t know	31.3 (29.7–33.1)	Reference		27.3 (25.7–29.0)	Reference	

Data are weighted. Analyses are unadjusted. High motivation to quit smoking: “I want to quit smoking a lot” vs. “other” (somewhat, a little, not at all)

*P*-value is for the main effects (omnibus test). *OR*, odds ratio; *CI*, confidence interval. Current frequency of cannabis use: (1) “daily cannabis use”; (2) “less than daily, but at least weekly/monthly cannabis use”; (3) “less than monthly (occasional) use and used it in the last year”; or (4) “no current cannabis use” (never used/quit using it/have not used it in the last year). Alcohol consumption was defined using the National Institute on Alcohol Abuse and Alcoholism’s definition (NIAAA), where levels of alcohol use are defined as “low risk” (≤ 4 drinks per occasion for men, ≤ 2 drinks per occasion for women) and “high risk” (≥ 5 drinks per occasion for men, ≥ 3 drinks per occasion for women, and ≥ 6 drinks on a single occasion at least once a month for men and women). All respondents were screened for past 30-day depressive symptomology using the two-question case finding instrument (TQI) for depression. Respondents who reported “yes” to both questions were classified as screening positive for depressive symptoms

Third, we ran three adjusted logistic regression models that tested (1) the contribution of the frequency of cannabis use, high-risk alcohol use (vs. low-risk/none), and depressive symptomology (vs. none) on motivation to quit smoking, (2) whether there were interaction effects between country and the independent variables, and (3) the 2- and 3-way interaction effects between the three independent variables. Thereafter, we ran four separate additional models with the data stratified by each country (Table 3). This was then repeated for the attempts to quit smoking outcome (Table 4). Finally, we tested whether motivation to quit smoking was associated with attempts to quit smoking, overall and by country using five separate adjusted logistic regression models (Table 5). We also tested whether there was an interaction effect between country and motivation to quit. All models adjusted for age, sex, education, income, race/ethnicity, and CPD. The overall models that included all respondents also adjusted for country of residence.

**Table 3:** The contribution of cannabis use, alcohol use, and depressive symptomology on motivation to quit smoking

	All respondents*		Australia (*n* = 1113)		Canada (*n* = 2069)		England (*n* = 2444)		USA (*n* = 1418)	
	% yes	aOR (95% CI)	% yes	aOR (95% CI)	% yes	aOR (95% CI)	% yes	aOR (95% CI)	% yes	aOR (95% CI)
Outcome: *‘I want to quit smoking a lot’* (n = 2301)	33.1%	—	38.8%	1.83 (1.45–2.31)	38.6%	1.81 (1.52–2.16)	25.8%	Reference	34.5%	1.52 (1.24–1.86)
Cannabis use		*p* = 0.53		*p* = 0.75		*p* = 0.72		*p* = 0.25		*p* = 0.11
Daily cannabis use	31.8%	0.91 (0.73–1.14)	34.4%	0.86 (0.35–2.16)	38.0%	1.00 (0.74–1.35)	25.8%	0.96 (0.57–1.61)	30.6%	0.76 (0.46–1.26)
Weekly/monthly use	31.6%	0.91 (0.70–1.17)	46.6%	1.44 (0.66–3.14)	36.8%	0.95 (0.67–1.34)	22.1%	0.78 (0.40–1.52)	31.0%	0.77 (0.46–1.30)
< monthly use (occasional)	30.2%	0.84 (0.65–1.10)	33.4%	0.83 (0.36–1.89)	42.6%	1.21 (0.85–1.72)	14.9%	0.48 (0.23–1.00)	22.9%	0.51 (0.29–0.91)
No current cannabis use	33.9%	Reference	37.8%	Reference	38.1%	Reference	26.7%	Reference	36.7%	Reference
Cannabis use*country interaction, *p*-value	*p* = 0.38									
Alcohol use		*p* = 0.02		*p* = 0.60		*p* = 0.043		*p* = 0.22		*p* = 0.03
High-risk	29.6%	0.81 (0.68–0.96)	39.6%	1.12 (0.73–1.71)	33.2%	0.75 (0.57–0.99)	22.8%	0.81 (0.57–1.13)	25.7%	0.61 (0.39–0.96)
Low-risk/none	34.3%	Reference	37.0%	Reference	39.8%	Reference	26.8%	Reference	36.0%	Reference
Alcohol use*country interaction, *p*-value	*p* = 0.27									
Depressive symptomology		*p* < 0.001		*p* = 0.008		*p* < 0.001		*p* = 0.17		*p* = 0.02
Yes	39.5%	1.46 (1.25–1.71)	47.7%	1.74 (1.16–2.61)	46.9%	1.64 (1.30–2.07)	28.7%	1.25 (0.91–1.71)	41.9%	1.52 (1.06–2.18)
No	30.9%	Reference	34.4%	Reference	35.0%	Reference	24.4%	Reference	32.2%	Reference
Depressive symptomology*country interaction, *p*-value	*p* = 0.46									

Data are weighted. All models adjusted for age, sex, income, education, race/ethnicity, and CPD. *The overall model also adjusted for country. *P*-value is for the main omnibus test. Outcome: I want to quit smoking a lot vs. other response. Wald = 5.06, *p* < 0.001. “% yes” is the weighted proportion who selected “I want to quit smoking a lot.” Current frequency of cannabis use: (1) “daily cannabis use”; (2) “less than daily, but at least weekly/monthly cannabis use”; (3) “less than monthly (occasional) use and used it in the last year”; or (4) “no current cannabis use” (never used/quit using it/have not used it in the last year). Alcohol consumption was defined using the National Institute on Alcohol Abuse and Alcoholism’s definition (NIAAA), where levels of alcohol use are defined as “low risk” (≤ 4 drinks per occasion for men, ≤ 2 drinks per occasion for women) and “high risk” (≥ 5 drinks per occasion for men, ≥ 3 drinks per occasion for women, and ≥ 6 drinks on a single occasion at least once a month for men and women). All respondents were screened for past 30-day depressive symptomology using the two-question case finding instrument (TQI) for depression. Respondents who reported “yes” to both questions were classified as screening positive for depressive symptoms

**Table 4:** The contribution of cannabis use, alcohol use, and depressive symptomology on attempts to quit smoking in the last year

	All respondents*		Australia (*n* = 1113)		Canada (*n* = 2069)		England (*n* = 2444)		USA (*n* = 1418)	
	%	aOR (95% CI)	%	aOR (95% CI)	%	aOR (95% CI)	%	aOR (95% CI)	%	aOR (95% CI)
Made a quit attempt (*n* = 2260)	29.1%	—	38.9%	1.96 (1.55–2.48)	33.7%	1.56 (1.31–1.87)	24.6%	Reference	24.1%	0.97 (0.79–1.21)
Cannabis use		*p* = 0.45		*p* = 0.75		*p* = 0.75		*p* = 0.051		*p* = 0.83
Daily cannabis use	28.5%	0.98 (0.78–1.24)	34.4%	0.86 (0.35–2.16)	34.3%	1.09 (0.80–1.48)	23.0%	0.92 (0.53–1.58)	24.5%	1.06 (0.65–1.75)
Weekly/monthly use	33.0%	1.22 (0.95–1.57)	46.6%	1.44 (0.66–3.14)	33.1%	1.03 (0.73–1.46)	37.7%	1.86 (1.06–3.28)†	24.2%	1.04 (0.58–1.88)
< monthly use (occasional)	28.3%	0.98 (0.74–1.29)	33.4%	0.83 (0.36–1.89)	37.0%	1.22 (0.84–1.79)	16.4%	0.60 (0.30–1.23)	19.0%	0.77 (0.41–1.43)
No current cannabis use	28.8%	Reference	37.8%	Reference	32.5%	Reference	24.5%	Reference	23.4%	Reference
Cannabis use*country interaction, *p*-value	*p* = 0.34									
Alcohol use		*p* = 0.004		*p* = 0.60		*p* = 0.037		*p* = 0.12		*p* = 0.25
High-risk	25.1%	0.77 (0.64–0.92)	39.6%	1.12 (0.73–1.71)	28.3%	0.74 (0.56–0.98)	21.1%	0.76 (0.55–1.07)	19.5%	0.76 (0.48–1.21)
Low-risk/none	30.4%	Reference	37.0%	Reference	34.7%	Reference	26.0%	Reference	24.1%	Reference
Alcohol use*country interaction, *p*-value	*p* = 0.91									
Depressive symptomology		*p* < 0.001		*p* = 0.008		*p* = 0.002		*p* = 0.01		*p* < 0.001
Yes	36.9%	1.63 (1.39–1.91)	47.7%	1.74 (1.16–2.61)	39.6%	1.47 (1.15–1.87)	30.3%	1.51 (1.10–2.07)	34.4%	2.01 (1.37–2.94)
No	26.4%	Reference	34.4%	Reference	30.9%	Reference	22.4%	Reference	20.7%	Reference
Depressive symptomology*country interaction, *p*-value	*p* = 0.40									

Data are weighted. All models adjusted for age, sex, income, education, race/ethnicity, and CPD. *The overall model also adjusted for country. *P*-value is for the main omnibus test. Outcome: made an attempt to quit smoking cigarette in the last year vs. did not make an attempt to quit smoking cigarettes in the last year/don’t know. Wald = 10.57, *p* < 0.001. Current frequency of cannabis use: (1) “daily cannabis use”; (2) ‘less than daily, but at least weekly/monthly cannabis use”; (3) “less than monthly (occasional) use and used it in the last year”; or (4) “no current cannabis use” (never used/quit using it/have not used it in the last year). Alcohol consumption was defined using the National Institute on Alcohol Abuse and Alcoholism’s definition (NIAAA), where levels of alcohol use are defined as “low risk” (≤ 4 drinks per occasion for men, ≤ 2 drinks per occasion for women) and “high risk” (≥ 5 drinks per occasion for men, ≥ 3 drinks per occasion for women, and ≥ 6 drinks on a single occasion at least once a month for men and women). All respondents were screened for past 30-day depressive symptomology using the two-question case finding instrument (TQI) for depression. Respondents who reported “yes” to both questions were classified as screening positive for depressive symptoms. †A Bonferroni adjustment for multiple comparisons was conducted and was not significant: aOR = 1.86 (95% CI, 0.87–3.98)

**Table 5:** The association between motivation to quit smoking and having made an attempt quit smoking in the past 12 months, overall and by country

	All respondents*		Australia (*n* = 1113)		Canada (*n* = 2069)		England (*n* = 2444)		USA (*n* = 1418)	
	%	aOR (95% CI)	%	aOR (95% CI)	%	aOR (95% CI)	%	aOR (95% CI)	%	aOR (95% CI)
Motivation to quit smoking		*p* < 0.001		*p* < 0.001		*p* < 0.001		*p* < 0.001		*p* < 0.001
A lot	54.6%	6.13 (5.25–7.17)	62.8%	5.86 (3.96–8.67)	57.4%	6.05 (4.73–7.74)	52.5%	6.30 (4.59–8.66)	47.1%	7.17 (5.03–10.20)
Not at all/a little/somewhat/don’t know	16.4%	Reference	22.3%	Reference	18.2%	Reference	14.9%	Reference	11.1%	Reference
Motivation to quit smoking*country interaction, *p*-value	*p* = 0.80									
Cannabis use		*p* = 0.23		*p* = 0.97		*p* = 0.87		*p* = 0.02		*p* = 0.83
Daily cannabis use	26.3%	1.02 (0.79–1.32)	32.4%	0.84 (0.38–1.85)	32.2%	1.10 (0.79–1.55)	20.4%	0.92 (0.51–1.69)	22.6%	1.25 (0.71–2.19)
Non-daily cannabis use	31.6%	1.32 (1.02–1.72)	34.9%	0.93 (0.42–2.06)	31.3%	1.06 (0.72–1.56)	38.5%	2.26 (1.29–3.99)	22.1%	1.21 (0.67–2.20)
Occasional cannabis use	26.9%	1.05 (0.78–1.42)	37.0%	1.03 (0.42–2.52)	33.3%	1.16 (0.77–1.76)	17.1%	0.74 (0.38–1.48)	19.3%	1.02 (0.56–1.87)
No current cannabis use	25.9%	Reference	36.4%	Reference	30.0%	Reference	21.7%	Reference	19.0%	Reference
Alcohol use		*p* = 0.03		*p* = 0.13		*p* = 0.15		*p* = 0.23		*p* = 0.81
High-risk	23.4%	0.81 (0.67–0.98)	30.6%	0.70 (0.45–1.11)	27.2%	0.80 (0.59–1.08)	19.5%	0.80 (0.56–1.16)	19.0%	0.94 (0.56–1.56)
Low-risk/none	27.5%	Reference	38.6%	Reference	31.9%	Reference	23.2%	Reference	20.0%	Reference
Depressive symptomology		*p* < 0.001		*p* = 0.01		*p* = 0.11		*p* = 0.03		*p* = 0.003
Yes	32.5%	1.49 (1.25–1.78)	46.4%	1.79 (1.13–2.83)	34.3%	1.25 (0.95–1.63)	27.1%	1.47 (1.04–2.07)	28.9%	1.90 (1.24–2.90)
No	24.4%	Reference	32.7%	Reference	29.5%	Reference	20.2%	Reference	17.7%	Reference

Data are weighted and adjusted for age, sex, income, education, race/ethnicity, and CPD. *The overall model also adjusted for country. *P*-value is for the main omnibus test. Outcome: made an attempt to quit smoking cigarette in the last year vs. did not make an attempt to quit smoking cigarettes in the last year/don’t know. Wald = 10.05, *p* < 0.001. Current frequency of cannabis use: (1) “daily cannabis use”; (2) “less than daily, but at least weekly/monthly cannabis use”; (3) “less than monthly (occasional) use and used it in the last year”; or (4) “no current cannabis use” (never used/quit using it/have not used it in the last year). Alcohol consumption was defined using the National Institute on Alcohol Abuse and Alcoholism’s definition (NIAAA), where levels of alcohol use are defined as “low risk” (≤ 4 drinks per occasion for men, ≤ 2 drinks per occasion for women) and “high risk” (≥ 5 drinks per occasion for men, ≥ 3 drinks per occasion for women, and ≥ 6 drinks on a single occasion at least once a month for men and women). All respondents were screened for past 30-day depressive symptomology using the two-question case finding instrument (TQI) for depression. Respondents who reported “yes” to both questions were classified as screening positive for depressive symptoms

## Results

This study included 7044 eligible adults who were smoking daily at the time of the 2020 survey (see Table 1). A majority of respondents were aged 40 + years (63.0%), White/English-speaking (85.0%), smoked daily for more than 10 years (67.4%), not currently using cannabis (71.3%), and did not report depressive symptoms (72.8%).

### Motivation to Quit Smoking and Attempts to Quit Smoking, Overall and by Country

Overall, 33.1% of adults reported that they wanted to quit smoking a lot, which differed significantly by country (*p* < 0.001). Adults in England were significantly less likely to report wanting to quit smoking a lot (25.8%) compared to adults in Australia (38.8%), Canada (38.6%), and the USA (34.5%) (Table 3).

Overall 29.1% of adults reported that they had made an attempt to quit smoking in the last year, which differed significantly by country (*p* < 0.001). Adults in Australia (38.9%) and Canada (33.7%) were significantly more likely than adults in England (24.6%) and the USA (24.1%) to have made a quit attempt in the past 12 months (Table 4).

### Prevalence of Daily Cannabis Use, High-Risk Alcohol Use, and Depressive Symptomology Among Adults Who Smoke Daily, Overall and by Country

Overall, 11.9% (95% CI, 10.7–13.1%) of adults reported daily cannabis use (highest in Canada, 17.7%, and lowest in Australia, 6.4%; Supplemental Table 2 presents all levels of cannabis use frequencies among adults who smoke daily, overall and by country); 23.1% (95% CI, 21.8–24.5%) reported high-risk alcohol use (highest in Australia, 30.0%, and lowest in the USA, 16.0%); and 27.4% (95% CI, 26.0–28.8%) reported depressive symptomology (highest in England, 29.5%, and lowest in the USA, 22.0%). Country of residence was independently associated with each of the three independent variables: (1) cannabis use (*p* < 0.001), high-risk alcohol use (*p* < 0.001), and depression (*p* = 0.0004) (Fig. 2).

### Contribution of Cannabis Use, High-Risk Alcohol Use, and Depressive Symptomology on Motivation to Quit Cigarette Smoking and Quit Attempts, Overall and by Country.

#### Cannabis Use

In the unadjusted models, there was no association with motivation to quit smoking and frequency cannabis use (*p* = 0.88); however, frequency of cannabis use was associated with making a quit attempt (*p* = 0.04), where those who were using cannabis on a weekly/monthly basis were more likely to have made an attempt to quit smoking (OR = 1.39, 95% CI, 1.10–1.76) compared to adults not using cannabis. Daily or less than monthly (occasional) cannabis use did not significantly differ from those who were not using cannabis (Table 2).

In the adjusted logistic regression models, the frequency of cannabis use was not associated with either outcome in the overall model or when data were stratified by country (Tables 3 and 4, respectively). There were no interactions between cannabis use and country on motivation to quit (*p* = 0.38) or quit attempts (*p* = 0.34).

#### High-Risk Alcohol Use

In the unadjusted models, high-risk alcohol use was associated with lower motivation to quit smoking (OR = 0.78, 95% CI, 0.66–0.92), but not with having made an attempt to quit smoking (OR = 0.91, 95% CI, 0.77–1.07) compared to those who reported low-risk alcohol consumption/no alcohol consumption (Table 2).

In the adjusted models, high-risk alcohol use was associated with lower motivation to quit smoking (aOR = 0.81, 95% CI, 0.68–0.96) and lower odds of having made an attempt to quit smoking (aOR = 0.77, 95% CI, 0.64–0.92) compared to those who reported low-risk alcohol consumption/no alcohol consumption (Tables 3 and 4, respectively). When the data were stratified by country, the association between high-risk alcohol use and lower motivation to quit was significant in Canada (*p* = 0.04) and the USA (*p* = 0.03), but was not significant in Australia (*p* = 0.60) or England (*p* = 0.22) (Table 3). The association between high-risk alcohol use and lower odds of making an attempt to quit smoking was only significant in Canada (*p* = 0.037), and not the other three countries (all *p* ≥ 0.05) (Table 4). There were no interactions between high-risk alcohol use and country on motivation to quit smoking (*p* = 0.27) or quit attempts (*p* = 0.91).

#### Depressive Symptomology

In the unadjusted models, depressive symptomology was associated with higher motivation to quit smoking (OR = 1.40, 95% CI, 1.20–1.62) and a greater likelihood of making an attempt to quit smoking (OR = 1.59, 95% CI, 1.37–1.85) compared to adults without depressive symptoms (Table 2).

In the adjusted models, depressive symptomatology remained significant for increased odds of higher motivation to quit smoking (aOR = 1.46, 95% CI, 1.25–1.71) and having made an attempt to quit smoking (aOR = 1.63, 95% CI, 1.39–1.91) (Tables 3 and 4, respectively). When the data were stratified by country, the association between depressive symptomology and higher motivation to quit was significant in Australia (*p* = 0.008), Canada (*p* < 0.001), and the USA (*p* = 0.02), but was not in England (*p* = 0.17) (Table 3). The association between depressive symptomology and a greater likelihood of making an attempt to quit smoking was significant in all four countries (all *p* < 0.05).

### Interaction Tests Between Independent Variables

There were no significant 2-way or 3-way interactions between cannabis use, high-risk alcohol use, and depressive symptomology on motivation to quit or quit attempts (see Supplemental Tables 1 and 2).

### The Association Between Motivation to Quit Smoking and Having Made an Attempt Quit Smoking in the Past 12 Months, Overall and by Country

Overall, adults who wanted to quit smoking a lot were significantly more likely to have reported a quit attempt in the past 12 months (aOR = 6.13, 95% CI, 5.25–7.17) than those who reported wanting to quit smoking a little/somewhat/not at all/don’t know. This relationship was significant across all countries. The addition of motivation to quit smoking to the model did not change the associations between quit attempts and cannabis use (*p* = 0.23), alcohol use (*p* = 0.03), or depressive symptomology (*p* < 0.001) (Table 5).

## Discussion

This international cross-sectional study assessed the independent and interaction effects of cannabis use, high-risk alcohol use, and depressive symptomology on motivation to quit cigarette smoking and attempts to quit among adults who smoked daily in Australia, Canada, England, and the USA. We found a third of adults in this study reported being highly motivated to quit smoking cigarettes, and slightly more than a quarter (29%) made an attempt to quit smoking in the last year. Cannabis use was not associated with motivation to stop smoking cigarettes or attempting to quit, either when analyzed as an individual factor or in conjunction with other factors. By contrast, high-risk alcohol use was associated with lower odds of being highly motivated to quit and quit attempts, and depressive symptomology was associated with increased odds for both outcomes. High-risk alcohol use was only associated with reduced odds of making a quit attempt in Canada. Depressive symptomology was associated with increased odds of quit attempts in all four countries. There were no significant 2- or 3-way interaction effects between cannabis use, alcohol use, and depressive symptoms.

Population studies generally show that motivation to quit smoking predicts quit attempts, but not the success of those attempts (Borland et al., 2010; de Granda-Orive et al., 2021; Pisinger et al., 2005; Ussher et al., 2016; Vangeli et al., 2011; West, 2004). When we added motivation to quit smoking cigarettes in our adjusted model, we found adults who were highly motivated to quit smoking reported having six times greater odds of reporting a recent quit attempt relative to those who were less motivated to quit. Based on previous research indicating that people motivated to quit smoking may not necessarily be more successful at maintaining abstinence during a quit attempt, tobacco treatment would likely benefit from focusing on other factors that predict staying quit rather than motivation (e.g., ways to increase self-efficacy, addressing and advising on how to manage both physiological and behavioral dependence on cigarettes/nicotine, the provision of cessation aids, cognitive behavioral counselling). Additionally, addressing other factors that may act as barriers to quitting, including substance use, dependence, and mental health, would likely be important during the treatment process. However, it is reassuring that cannabis use was not associated with reduced motivation or quit attempts, considering that many jurisdictions have legalized or plan to legalize non-medical cannabis.

When we examined motivation to quit overall and then by country, we found that only one-third of adults who smoked daily reported being highly motivated to quit; however, adults in England were far less likely to report this relative to the other three countries. Additionally, only 29% of adults reported making a quit attempt, with lower odds of quit attempts in England and the USA relative to Canada and Australia. It is not clear to us why country-specific differences were observed for both interest in quitting and quit attempts, even after adjustment for sociodemographic differences; however, it has been found that adults who smoke in England fail to report a substantial proportion of unsuccessful quit attempts, particularly if they last a short time and/or if the attempt was made without support (Berg et al., 2010; Perski et al., 2022). Similar to our study, a previous study using ITC Project data in the four countries included herein assessed quit attempts between 2002 and 2005 and found that adults who smoked were less likely to have attempted to quit in England relative to the other three countries; however, among those who made a quit attempt, they were more likely to have used cessation support and stayed quit (Gibson et al., 2010). Thus, the lower quit attempt rate reported in England may be an artifact of using a quit aid, and may go largely unreported among those who did not use one. Further research is warranted to explore other factors that might account for the differences observed between countries, potentially related to COVID restrictions, policies affecting access to tobacco products, and access to healthcare, which might impact interactions with health care providers.

In contrast to past studies suggesting that more frequent cannabis use may result in diminished motivation to quit smoking and attempts to quit (Strong et al., 2018; Twyman et al., 2016), we did not find evidence of this in the current study. Interestingly, quit attempts differed very little between those who used cannabis daily compared to those who did not use cannabis at all. However, further investigation is required using longitudinal data to assess subsequent success in achieving abstinence, while considering other potential modifiers of cannabis use. For example, cravings for tobacco may vary for some individuals following cannabis use (sequential use), while co-administration (mixing tobacco and cannabis) of products could lead to pronounced impacts on tobacco use outcomes; measurement of co-use patterns would allow further exploration of these influences (Hindocha & McClure, 2021). The mode of administration of cannabis products used (inhaled vs. oral consumption) and reasons for using cannabis (medical vs. non-medical) may also play a role in the relationship between substances, as well as underlying neuropsychological relationships supporting co-use (Akbar et al., 2019; Jayakumar et al., 2021; Rabin & George, 2015; Twyman et al., 2016; Volkow et al., 2016). Furthermore, the legalization of cannabis, along with the growing diversity of cannabis products, should be explored to assess whether increased access to cannabis impacts tobacco use. However, a recent study by Chu et al. found that cannabis consumers who used tobacco were lower in legal jurisdictions in Canada and the USA, despite higher prevalence of cannabis use. Edible use was inversely associated with co-use, suggesting that edible use does not appear to be associated with tobacco use (Chu et al., 2023).

Our findings showed that overall, high-risk alcohol use was associated with decreased odds of being highly motivated to quit smoking and making a quit attempt. When we analyzed the data by country, high-risk alcohol use was associated with lower rates of motivation to quit in the USA and Canada, but only in Canada was high-risk alcohol use associated with reduced odds of attempting to quit smoking. Perhaps differences between countries in drinking cultures (e.g., wet or dry cultures, Savic et al., 2016), attitudes toward alcohol use, patterns of risky alcohol consumption, drink sizes and strengths, and types of alcohol typically consumed (beer, wine, vs. hard liquor) may have differing consequences when making between-country comparisons on substance use behaviors. Additionally, low statistical power when data were stratified by country may explain the failure to find an effect in the USA and England, where similar to Canada, a lower proportion of people who engaged in high-risk alcohol use made a quit attempt. However, because alcohol and tobacco co-use is so common, addressing alcohol use during treatment for smoking cessation may benefit many individuals who perceive that their alcohol use is a barrier to stopping smoking (Toll et al., 2012, 2015). It should also be noted that even though there was no correlation between high-risk alcohol use and trying to quit smoking in Australia and England, we found that risky alcohol consumption was most prevalent in these two countries. The high percentage of adults who smoke daily and engage in risky alcohol use in these two countries should be addressed by healthcare professionals regardless of whether it has an impact on tobacco use, as alcohol consumption alone poses significant risks (CDC, 2022), and there are adverse synergistic effects when alcohol and tobacco are used together (Hart et al., 2010; IARC, 2004; Prabhu et al., 2014). Indeed in Canada, new guidance urges medical practitioners to regularly screen for high-risk drinking and alcohol use disorder (Wood et al., 2023).

Our findings corroborate other studies reporting that people with depression are highly motivated to quit and more likely to try to quit than those without depression, which was consistent across all four countries. There could be several reasons for this, including higher rates of engaging with healthcare professionals and receiving advice about stopping smoking, receiving information that quitting smoking improves mental health and mood, and concerns over the impact of smoking on short- and long-term physical health. However, only two in five adults who self-reported depressive symptoms reported attempting to quit smoking in the past year. Thus, clinicians should routinely screen for depression among those who smoke cigarettes and strongly recommend quitting, as well as assess individual barriers to remaining abstinent after trying to quit among this sub-population, including addressing any concerns about worsening depression or anxiety after a quit attempt, and treating withdrawal symptoms during a quit attempt. Coping techniques should also be routinely provided to reduce relapse rates. The importance of behavioral counselling as part of a quit attempt, which includes individualized coping strategies, should be strongly encouraged and readily available, given such counselling is rarely used by adults who do make a quit attempt (Gravely et al., 2021). Further, cohort studies are warranted to examine whether other mental health conditions (e.g., schizophrenia, bipolar, anxiety disorders, major depressive disorder) may interact with cannabis and alcohol use, which may diminish smoking cessation activity.

We did not find any interactions between cannabis use, depressive symptomology, and high-risk alcohol use on quit motivation or quit attempts; however, understanding just how these factors interact is challenging. There are several linked biological mechanisms, common risk factors, shared social and environmental influences, and genetic vulnerabilities that make disentangling the impact of these factors difficult. Better understanding of the role of co-occurring substance use and mental health on tobacco quit motivation, quit attempts, and cessation will have important tobacco treatment implications. The population of those who are smoking cigarettes continues to change, largely affecting those who use other substances, have mental health disorders, and/or belong to historically minoritized racial and ethnic groups or sexual and gender minorities (ASH UK, 2019; Carroll et al., 2019; CDC, 2023; Chaiton & Callard, 2019; Glover et al., 2020). Further, those with psychiatric and co-occurring substance use tend to have greater nicotine dependence and poorer cessation outcomes (Han et al., 2023; Sharma et al., 2016). Therefore, tobacco treatment guidelines should incorporate elements tailored to the individual based on additional barriers to cessation that may exist, which will be informed by work assessing the individual and synergistic impact of co-occurring factors.

Although this is a large study with a representative sample of adults who smoke cigarettes daily from four countries, there are limitations to consider. First, this is a cross-sectional study; therefore, the directional relationship between depression, cannabis use, alcohol use, and cigarette smoking cannot be determined with the outcomes measured. Second, the sample was limited to adults who smoke cigarettes daily, rather than more inclusive definitions of those who smoke non-daily, which may partially contribute to discrepancies in our findings against the broader literature from other national surveys. Furthermore, people who successfully quit smoking in the past year (and were smoking daily previous to their quit attempt) were excluded from this study (e.g., because they were not asked about nicotine dependence or motivation to quit smoking). Thus, the current sample included herein were those who did not attempt to stop smoking, or attempted to quit but were unsuccessful, which could have implications (e.g., they were more dependent on nicotine, they may have been less motivated to do so, may have been more likely to have used cannabis and/or alcohol and/or have depressive symptoms). Cohort studies are needed to validate quit successes using a prospective design to test whether cannabis use, alcohol misuse, and/or depressive symptoms impact smoking cessation across time. Third, this study was conducted just prior to and during the first few months of the COVID-19 pandemic which could have impacted substance use and mental health. Findings may have differed before or after the pandemic, thus requiring further investigation. Forth, the outcomes could be subject to recall bias. Finally, population estimates of quit attempts based on retrospective data may be substantially underestimated, particularly if they only lasted a short time or cessation support was not used during the quit attempt.

## Conclusions

Overall, just over a quarter of adults who smoked daily reported making a recent quit attempt, and most were not highly motivated to quit. Cannabis use was not associated with motivation to quit smoking or quit attempts. High-risk alcohol use was significantly associated with decreased odds of motivation to quit and making a quit attempt, but this varied by country. Depressive symptomology and higher motivation to quit smoking were associated with greater odds of making a quit attempt, which was consistent across all four countries. Understanding how alcohol, cannabis, and mental health interact and affect tobacco use, motivation to quit, attempts to quit, and successful cessation is challenging as each factor may individually or synergistically pose a barrier to treatment. Tobacco cessation should be evaluated using longitudinal study designs to determine factors that may reduce quitting activity.

## Supplementary Information

Below is the link to the electronic supplementary material.

Supplementary file1 (DOCX 28 kb)

## Data Availability

The data are jointly owned by a third party in each country that collaborates with the International Tobacco Control Policy Evaluation (ITC) Project. Data from the ITC Project are available to approved researchers 2 years after the date of issuance of cleaned data sets by the ITC Data Management Centre. Researchers interested in using ITC data are required to apply for approval by submitting an International Tobacco Control Data Repository (ITCDR) request application and subsequently to sign an ITCDR Data Usage Agreement. The criteria for data usage approval and the contents of the Data Usage Agreement are described online (http://www.itcproject.org). The authors of this paper obtained the data following this procedure. This is to confirm that others would be able to access these data in the same manner as the authors. The authors did not have any special access privileges that others would not have.
