# Supplementary material for: Associations of Cannabis Use, High-Risk Alcohol Use, and Depressive Symptomology with Motivation and Attempts to Quit Cigarette Smoking Among Adults: Findings from the 2020 ITC Four Country Smoking and Vaping Survey
Source: Int J Ment Health Addict. 2023 Dec 14;23(3):2021–45. doi: 10.1007/s11469-023-01214-8 (PMC12279565; doi:10.1007/s11469-023-01214-8)
Supplement: Supplementary file 1 — Supplementary file1 (DOCX 28 kb) [file 11469_2023_1214_MOESM1_ESM.docx]

**Supplemental Table 1.** Prevalence of the frequency of cannabis use among adults who smoke daily in Australia, Canada, England and the United States (N=7,044)

|  | **Canada**  n=2,069 | **United States**  n=1,418 | **England**  n=2,444 | **Australia**  n=1,113 | **All***  N=7,044 |
| --- | --- | --- | --- | --- | --- |
| **Weighted %**  **(95% CI)** | | | | | |
| **Frequency of cannabis use** |  |  |  |  |  |
| Daily | 17.7%  (15.8%-19.7%) | 16.3%  (13.4%-19.2%) | 8.4%  (6.5%-10.2%) | 6.4%  (4.1% - 8.7%) | 11.9%  (10.7%-13.1%) |
| Weekly/monthly use | 12.0%  (10.4%-13.6%) | 8.6%  (6.7%-10.5%) | 5.8%  (4.5%-7.1%) | 6.5%  (4.3%-8.6%) | 8.2%  (7.3%-9.1%) |
| <Monthly use | 10.2%  (8.7%-11.7%) | 6.1%  (4.6%-7.5%) | 4.8%  (3.5%-6.2%) | 7.4%  (4.8%-9.9%) | 6.9%  (6.1%-7.8%) |
| None | 60.1%  (57.6%-62.6%) | 69.0%  (65.7%-72.4%) | 81.0%  (78.6%-83.4%) | 79.7%  (76.1%-83.4%) | 73.0%  (71.4%-74.5%) |

Weighted descriptive statistics (‘proc surveyfreq’) were used to derive conditional estimates. Current frequency of cannabis use: (1) ‘daily cannabis use’; (2) ‘less than daily, but at least weekly/monthly cannabis use’; (3) ‘less than monthly (occasional) use and used it in the last year’; or (4) ‘no current cannabis use’ (never used/quit using it/have not used it in the last year).

**Supplemental Table 2.** Two-way interaction effects between cannabis use, high-risk alcohol use and depressive symptomology

| **Two-way interaction test on motivation to quit smoking** | | | | |
| --- | --- | --- | --- | --- |
| **Effect** | **F Value** | **Num DF** | **Den DF** | **Pr > F** |
| High-risk alcohol use (ALC) | 1.26 | 1 | 7008 | 0.26 |
| Frequency of cannabis use (CAN) | 0.29 | 3 | 7006 | 0.83 |
| Depressive symptoms (DEP) | 7.3 | 1 | 7008 | 0.007 |
| Age group | 0.06 | 1 | 7008 | 0.80 |
| Sex | 3.04 | 1 | 7008 | 0.08 |
| Country | 15.55 | 3 | 7006 | <.0001 |
| Income | 0.38 | 3 | 7006 | 0.77 |
| Education | 0.31 | 2 | 7007 | 0.73 |
| Race/ethnicity | 4.05 | 1 | 7008 | 0.04 |
| Cigarettes smoked per day | 0.86 | 2 | 7007 | 0.42 |
| **ALC*CAN** | **0.73** | **3** | **7006** | **0.53** |
| **ALC*DEP** | **0.26** | **1** | **7008** | **0.61** |
| **CAN*ALC** | **1.03** | **3** | **7006** | **0.38** |
| **Two-way interaction test on past year quit attempts** | | | | |
| **Effect** | **F Value** | **Num DF** | **Den DF** | **Pr > F** |
| High-risk alcohol use (ALC) | 3.51 | 1 | 7008 | 0.06 |
| Frequency of cannabis use (CAN) | 1.09 | 3 | 7006 | 0.35 |
| Depressive symptoms (DEP) | 10.34 | 1 | 7008 | 0.001 |
| Age group | 20.19 | 1 | 7008 | <.0001 |
| Sex | 1.01 | 1 | 7008 | 0.31 |
| Country | 17.36 | 3 | 7006 | <.0001 |
| Income | 3.68 | 3 | 7006 | 0.012 |
| Education | 3.19 | 2 | 7007 | 0.041 |
| Race/ethnicity | 9.04 | 1 | 7008 | 0.003 |
| Cigarettes smoked per day | 7.07 | 2 | 7007 | 0.0009 |
| **ALC*CAN** | **0.68** | **3** | **7006** | **0.57** |
| **ALC*DEP** | **0.74** | **1** | **7008** | **0.39** |
| **CAN*ALC** | **1.81** | **3** | **7006** | **0.14** |

All variables are categorical. Current frequency of cannabis use: (1) ‘daily cannabis use’; (2) ‘less than daily, but at least weekly/monthly cannabis use’; (3) ‘less than monthly (occasional) use and used it in the last year’; or (4) ‘no current cannabis use’ (never used/quit using it/have not used it in the last year). Alcohol consumption was defined using the National Institute on Alcohol Abuse and Alcoholism’s definition (NIAAA), where levels of alcohol use are defined as “low risk” (≤4 drinks per occasion for men, ≤2 drinks per occasion for women) and “high risk” (≥5 drinks per occasion for men, ≥3 drinks per occasion for women, and ≥6 drinks on a single occasion at least once a month for men and women). All respondents were screened for past 30-day depressive symptomology using the two-question case finding instrument (TQI) for depression. Respondents who reported ‘yes’ to both questions were classified as screening positive for depressive symptoms. Cigarettes smoked per day: ‘1-10’, ’11-20’, or ‘21+’.

**Supplemental Table 3.** Three-way interaction effects between cannabis use, high-risk alcohol use and depressive symptomology

| **Three-way interaction test on motivation to quit smoking** | | | | | |
| --- | --- | --- | --- | --- | --- |
| **Effect** | **F Value** | | **Num DF** | **Den DF** | **Pr > F** |
| High-risk alcohol use (ALC) | 0.95 | | 1 | 7008 | 0.33 |
| Frequency of cannabis use (CAN) | 0.32 | | 3 | 7006 | 0.81 |
| Depressive symptoms (DEP) | 7.64 | | 1 | 7008 | 0.006 |
| Age group | 0.05 | | 1 | 7008 | 0.82 |
| Sex | 3.21 | | 1 | 7008 | 0.07 |
| Country | 16 | | 3 | 7006 | <.0001 |
| Income | 0.36 | | 3 | 7006 | 0.78 |
| Education | 0.34 | | 2 | 7007 | 0.71 |
| Race/ethnicity | 4.31 | | 1 | 7008 | 0.038 |
| Cigarettes smoked per day | 0.82 | | 2 | 7007 | 0.44 |
| **ALC*CAN** | **1.26** | | **3** | **7006** | **0.29** |
| **ALC*DEP** | **0.31** | | **1** | **7008** | **0.58** |
| **CAN*ALC** | **0.99** | | **3** | **7006** | **0.40** |
| **ALC*CAN*DEP** | **1.95** | | **3** | **7006** | **0.12** |
| **Three-way interaction test on past year quit attempts** | | | | | |
| **Effect** | | **F Value** | **Num DF** | **Den DF** | **Pr > F** |
| High-risk alcohol use (ALC) | | 3.04 | 1 | 7008 | 0.081 |
| Frequency of cannabis use (CAN) | | 1.03 | 3 | 7006 | 0.38 |
| Depressive symptoms (DEP) | | 10.82 | 1 | 7008 | 0.001 |
| Age group | | 20.36 | 1 | 7008 | <0.0001 |
| Sex | | 0.98 | 1 | 7008 | 0.32 |
| Country | | 17.45 | 3 | 7006 | <0.0001 |
| Income | | 3.69 | 3 | 7006 | 0.011 |
| Education | | 3.21 | 2 | 7007 | 0.041 |
| Race/ethnicity | | 9.11 | 1 | 7008 | 0.003 |
| Cigarettes smoked per day | | 7.05 | 2 | 7007 | 0.001 |
| **ALC*CAN** | | **0.84** | **3** | **7006** | **0.47** |
| **ALC*DEP** | | **1.8** | **1** | **7008** | **0.18** |
| **CAN*ALC** | | **1.03** | **3** | **7006** | **0.38** |
| **ALC*CAN*DEP** | | **0.38** | **3** | **7006** | **0.77** |

All variables are categorical. Current frequency of cannabis use: (1) ‘daily cannabis use’; (2) ‘less than daily, but at least weekly/monthly cannabis use’; (3) ‘less than monthly (occasional) use and used it in the last year’; or (4) ‘no current cannabis use’ (never used/quit using it/have not used it in the last year). Alcohol consumption was defined using the National Institute on Alcohol Abuse and Alcoholism’s definition (NIAAA), where levels of alcohol use are defined as “low risk” (≤4 drinks per occasion for men, ≤2 drinks per occasion for women) and “high risk” (≥5 drinks per occasion for men, ≥3 drinks per occasion for women, and ≥6 drinks on a single occasion at least once a month for men and women). All respondents were screened for past 30-day depressive symptomology using the two-question case finding instrument (TQI) for depression. Respondents who reported ‘yes’ to both questions were classified as screening positive for depressive symptoms. Cigarettes smoked per day: ‘1-10’, ’11-20’, or ‘21+’
